# The Report of the Medical Officer to the Local Government Board

**Published:** 1917-02-03

**Authors:** 


### The Hospital

The Workers' Newspaper of Administrative Medicine and Institutional Life, Administration, National Insurance and Health.

No. 1600, Vol. LXI. SATURDAY, FEBRUARY 3, 1917. PRICE ONE PENNY.

### THE REPORT OF THE MEDICAL OFFICER TO THE LOCAL GOVERNMENT BOARD.

The Annual Report for 1915-16 of the Medical Officer to the Local Government Board (Dr. Arthur Newsholme) has just been issued, and in spite of the stress of war conditions contains almost as much information and careful thinking as usual. Naturally, the war and the special problems which it creates for health authorities in this country are taken prominently into consideration; so much so that with regard to some of the infectious diseases (measles and enteric fever, for instance) the notifications for the civil population and for the military are given in separate tables. In regard to enteric it is most satisfactory to note that notwithstanding some prevalence of this group of diseases in one at least of the theatres of war, its steady decline amidst the civilian population continues undisturbed; and Dr. Newsholme is sanguine enough to express the view that at no very distant date it will become as rare as the almost extinct typhus and cholera now are in these islands. Incidentally, a small outbreak at Reading took place, investigation of which showed that the water supply was probably being contaminated from a local hospital which had admitted cases of paratyphoid fever from overseas.

Those who read our recent article on the danger of smallpox arising through the war (The Hospital, January 13, 1917, p. 301) will be interested in what the report has to say on this topic. The Medical Officer is very emphatic as to the real danger of the introduction of smallpox into this country from abroad, and as to the lines of defence which can best be opposed to any such invasion. The importance of early diagnosis and immediate notification are pointed out, and also those of isolation, disinfection, and the vaccination of contacts. As regards hospital administration, the official policy is thoroughgoing and comprehensive, as several local authorities have lately been reminded. Dr. Newsholme is very definite on this matter, and all the more so because of the decline in vaccination and the consequent extra vulnerability of the population at large to a disastrous epidemic. He lays it down that in every district hospital accommodation for isolating the first cases should at once be available, and that further hospital accommodation should be available at short notice. Such smallpox hospitals should be as remote from inhabited districts as is practicable, and no other disease should be treated either in the same buildings or even on the same site. The hospital site should be adequately fenced, to prevent unauthorised communication between the occupants of the hospital and persons outside. There is nothing of novelty in these precautionary measures; but it is significant of the serious view which the Board's Medical Officer takes that they are put so prominently forward at the present time. As a matter of fact, two or three small outbreaks did occur in the year under review, which could be traced to imported infection: fortunately, they were detected early and overtaken at once by prompt action before the contagion became widely disseminated.

Tuberculosis and cerebrospinal meningitis receive a fair measure of attention, as also does maternity and child welfare. The most comprehensive monograph in the report, however, is that of Dr. Bruce Low on the Epidemiology of Acute Anterior Poliomyelitis, the disease which devastated New York last year.

Dr. Low's account was evidently drawn up before the full statistics of that outbreak were available, and before the latest views based upon the experience then gained had obtained publicity. He takes little account, for instance, of the hypothesis that paralysis is merely an occasional symptom of this particular disease; and that the majority of the cases are never recognised as poliomyelitis at all, because paralysis does not supervene. That, after all, is not strictly an epidemiological aspect of this still mysterious disease, to which aspect alone Dr. Low addresses himself. It is a curious fact that as recently as 1886 the then standard text-book on "Geographical and Historical Pathology," by Hirsch, made no mention at all of this disease, which has certainly been widely disseminated, and recognised by physicians, for at least a century. For his very comprehensive survey of its frequency in every quarter of the civilised world, Dr. Bruce Low deserves to be highly complimented.

